# TOSCA: an automated Tumor Only Somatic CAlling workflow for somatic mutation detection without matched normal samples

**DOI:** 10.1093/bioadv/vbac070

**Published:** 2022-09-26

**Authors:** Marcello Del Corvo, Saveria Mazzara, Stefano A Pileri

**Affiliations:** Division of Haematopathology, IEO, European Institute of Oncology IRCCS, Milan 20141, Italy; Division of Haematopathology, IEO, European Institute of Oncology IRCCS, Milan 20141, Italy; Division of Haematopathology, IEO, European Institute of Oncology IRCCS, Milan 20141, Italy

## Abstract

**Motivation:**

Accurate classification of somatic variants in a tumor sample is often accomplished by utilizing a paired normal tissue sample from the same patient to enable the separation of private germline mutations from somatic variants. However, a paired normal sample is not always available, making a reliable somatic variant calling more challenging. *In silico* screening of variants against public or private databases and other filtering approaches are often used in absence of a paired normal sample. Nevertheless, difficulties in performing a tumor-only calling with sufficient accuracy and lack of open-source software have limited their applications in clinical research.

**Results:**

To address these limitations, we developed TOSCA, the first automated tumor-only somatic calling workflow in whole-exome sequencing and targeted panel sequencing data which performs an end-to-end analysis from raw read files, via quality checks, alignment and variant calling to functional annotation, databases filtering, tumor purity and ploidy estimation and variant classification. Application of our workflow to tumor-only data provides estimates of somatic and germline variants that are consistent with results from paired analyses.

**Availability and implementation:**

TOSCA is a Snakemake-based workflow and freely available at https://github.com/mdelcorvo/TOSCA.

**Supplementary information:**

Supplementary data are available at *Bioinformatics Advances* online.

## 1 Introduction

A key issue cancer researchers currently face is the challenge of calling somatic mutations with enough reliability when analyzing cancer genomes. The identification of such driver changes, especially when they are located within key genes or DNA regulatory elements, is crucial in our understanding of cancer (Marx, 2002). In most cases, this is achieved by directly comparing genomic sequences from a tumor and matched normal sample of a given cancer patient. This allows subtraction of the germline variants shared by all cells in an individual, leaving only acquired somatic mutations (Li *et al*., 2014). However, sequencing a patient matched normal specimen in clinical diagnostic laboratories may often be difficult due to high cost of double sequencing and lack of availability of normal samples for some type of tumors. While the use of assays that analyze unmatched tumors is generally sufficient to identify cancer mutational hot spots (Boland *et al*., 2015), for more comprehensive assays, it is necessary to distinguish algorithmically between somatic mutations and germline status. Current approaches commonly involve *in silico* filtering of variants against multiple information sources, including laboratories and public databases (Hiltemann *et al*., 2015). Recently developed tools additionally include allele-specific copy number, allowing accurate posterior probabilities estimation for all possible somatic and germline genotypes (Riester *et al.*, 2016).

In this study, we capitalize on this knowledge and propose a modular Tumor Only Somatic CAlling (TOSCA) workflow, which performs an end-to-end whole-exome / targeted sequencing analysis, including both preprocessing and downstream steps, with focus on somatic status predictions. More specifically, it allows users to easily perform quality assessment, adapter trimming, genome alignment, variant calling, sequencing metrics and database annotations. The identification of somatic status leverages two different approaches: an optimized variant filtration strategy and an R package for detection of somatic status via tumor purity and ploidy estimation. As to our knowledge, TOSCA is the first modular open-source framework for tumor-only somatic calling, especially in diagnostic settings.

## 2 Software description

### 2.1 Implementation details

**Fig. 1.**
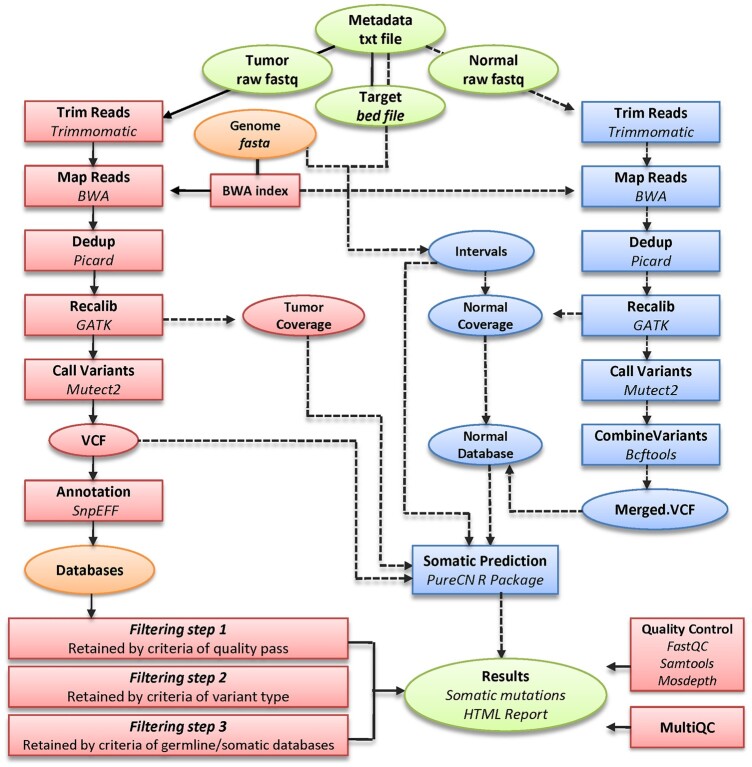
Simplified directed acyclic graph (DAG) of the TOSCA workflow. Oval shapes are referred to data (input, output and intermediate files), while rectangles to processes. Raw input/output files are depicted on the top and the botton of the graph by green ovals, respectively, while files independently downloaded by TOSCA are represented as orange ovals (reference genome and databases). Red blocks on the left are mandatory rules, while dashed lines and blue blocks on the right are optional rules, controlled in the configuration file. By default only mandatory rules are executed

TOSCA is implemented using the Snakemake management system (Köster and Rahmann, 2012) and conda environments. Snakemake ensures a well-controlled and scalable execution of an end-to-end analysis of whole-exome sequencing (WES) and targeted panel sequencing (TS) data, starting from one or more fastq files with raw sequencing reads representing distinct samples, either single-end or paired-end. A full description of different software implemented in TOSCA is given in Supplementary File S1, while a schematic representation of the whole workflow is given in Figure 1.

In the core step of TOSCA variants are annotated and classified based on their status. The workflow can be executed in the absence of any kind of normal control (‘pure’ tumor-only mode) or with some normal specimens (‘hybrid’ mode). In the first case, TOSCA activates a custom tumor-only filtration strategy inspired by a decision tree filtration algorithm developed by Sukhai *et al*. (2019). Our algorithm is designed in three phases and works in a similar way: first phase includes quality filtration based on quality pass or variant type (non-synonymous) criteria. Secondly, variants that exceeded the first step were marked as germline if they were present in any of the four germline variant databases at a minor allele frequency of 1% and labeled as somatic if they were found in the COSMIC database even if also found in a germline population database. Finally, in the third phase, variants were labeled germline if they were present as a benign or likely benign variants in ClinVar database. More details about filtration process can be found in Supplementary File S1. Our workflow can also be implemented in ‘hybrid’ mode by providing unmatched normal samples in the configuration file. TOSCA in this case re-runs all the steps including custom tumor-only filtration strategy and produces same outputs but in addiction, for each sample, activates a tumor purity and ploidy estimation via R package PureCN (Riester *et al.*, 2016). This in turn yields a parallel assessment of somatic status that possibly outperform previous one in terms of accuracy. In comparison with previous approach, PureCN is also able to deal with tumor-only WES data, as shown by Oh *et al*. (2020). The results obtained by these authors were highly concordant with matched normal WES data demonstrating that is possible to obtain reliable somatic mutational signatures also from whole-exome data in absence of normal tissue controls.

Other tools can be easily exchanged for those listed above by modifying the Snakefile and/or the template analysis code.

### 2.2 Input and output file

In order to run TOSCA, only two files must be prepared: a metadata with paths of FASTQ raw data and a configuration file with all the parameters required for the computation. First, the user is prompted to choose which version of the human genome to use as reference between hg19 and hg38. It is possible to set additional thresholds and parameters, including variant allele fraction and sequencing depth. Default and suggested cutoffs for both WES and TS data are provided in Supplementary File S1. Tumor-only filtering also requires queries against multiple databases. TOSCA relies on the following four germline population databases: 1000 Genomes phase 3, Exome Sequencing Project (ESP; ESP6500SI-V2 dataset of Exome Variant Server, National Heart, Lung, and Blood Institute Grand Opportunity Exome Sequencing Project, Seattle, WA, USA), Exome Aggregation Consortium version r1 (ExAC) and Single-Nucleotide Polymorphism Database (dbSNP). Variants are also tested against COSMIC, a somatically acquired mutations databases and against the germline/somatic database ClinVar (National Center for Biotechnology Information ClinVar). Overall, the information in these databases is limited by variable accuracy. For example, dbSNP, despite being considered a source of germline variants, contains also pathogenic variants, whereas COSMIC contains also some germline variants. Because of the relatively poor ability of dbSNP to correctly classify germline variants in comparison for example with ExAC, these databases have been assigned a different priority within tumor-only filtration strategy.

When the workflow starts, TOSCA automatically downloads all the necessary data from Ensembl ftp server, including reference genome, annotations and databases. This operation may require some time, mainly depending on connection speed, but it is usually done only once at the first use of the workflow and won’t be repeated until the user decides to update their environment. The check inputs rule in the Snakefile can be also executed to make sure all the input data and the parameters in the configuration file have been correctly specified.

The output files together with benchmark and log files for each block are stored in a directory specified by user in the configuration file. The final result of somatic calling is combined with all information retrieved from database and annotation files and saved as text file together with an html report resulting from MultiQC analysis (Ewels *et al*., 2016).

## 3 Validation

### 3.1 Targeted analysis validation

We selected an independent publicly available TS dataset of tumor and matched normal from a cohort of 16 patients affected by T-cell lymphoblastic lymphoma (T-LBL), as first case of our benchmarking study. TS was performed with a capture-based technique from Agilent Technologies and a targeted panel of 78 genes, with probes designed to cover all the coding regions of the genes. Raw sequencing reads were downloaded from the Sequence Read Archive (SRA) under the accession numbers PRJEB36436 (Khanam *et al*., 2021). Each read was aligned to the human reference genome GRCh38 (hg38). The cohort was divided into two groups: 10 tumor samples were used for tumor-only somatic variant calling, while 6 normal samples were used to build a normal database for coverage normalization, a step required for tumor purity and ploidy estimation. The gold standard origin of a mutation was obtained by running the same pre-processing step of TOSCA for both germline and tumor samples. Single-nucleotide variants were then called from paired BAM files with the same software but in ‘tumor with matched normal mode’ and marked by the following rules: whenever a variant appeared in the matched normal with significant allele frequency (>5%), it was considered germline, and tumor-only variants were called somatic. Overall, 10 of 16 paired samples were analyzed with both pure and hybrid tumor-only pipeline and validated with tumor-normal workflow while as for the remaining 6, germline side were used to build a database of unmatched samples for tumor purity and ploidy estimation. A total of 655 unique variants were detected and evaluated, including 57% (*N* = 376) germline and 43% (*N* = 279) somatic according to gold standard (Supplementary Table S1). TOSCA produced somatic versus germline calls for 100% of variants in the T-LBL samples, and among these calls, in pure tumor only mode somatic and germline variants were correctly classified with sensitivity and specificity values of 91% and 88%, respectively.

To demonstrate the importance of taking into account the genome-wide copy number profile for somatic/germline prediction, we applied TOSCA to the validation dataset also in hybrid mode by incorporating into the analysis a set of unmatched germline samples and again comparing results to gold standard list of mutations. In hybrid mode, TOSCA reached 96% for both sensitivity and specificity parameters, with an increase in accuracy of 4% and 7%, respectively, in comparison to previous analysis (Supplementary Table S1).

### 3.2 Whole-exome analysis validation

To further assess performance of the pipeline and demonstrate that the TOSCA workflow can obtain good performance also with WES data when the ‘hybrid’ mode is activated, we used data provided by the Somatic Working Group of SEQC-II Consortium, described in the Fang *et al.* (2021) article. They developed a tumor-normal reference sample using a normal B-lymphocyte cell line (i.e. HCC1395BL) and a triple-negative breast cancer cell line (i.e. HCC1395) from the same donor. Raw sequencing reads were downloaded from the SRA under the accession numbers SRP162370 (Fang *et al.*, 2021). Each read was aligned to the human reference genome GRCh38 (hg38). The dataset is based on 12 high coverage sequencing replicates for both HCC1395BL and HCC1395. The performance of TOSCA workflow was evaluated using sensitivity and precision metrics obtained by comparing the calls to corresponding truth set, which has been generated using multiple high coverage sequencing replicates and various combinations of data analysis algorithms, including six variant callers. We used the high confidence set which comprises 1089 SNVs and 57 Indels. As expected, when TOSCA is validated in a ‘pure’ tumor-only mode by using only tumor HCC1395 samples, WES analysis showed the lowest sensitivity and specificity of SNV detection (69% and 75% respectively). On the contrary, by incorporating normal HCC1395BL replicates as a pool of normal and taking into account the genome-wide copy number profile, somatic and germline variants were classified with higher accuracy (95.7% and 94.9%, respectively) (Supplementary Table S1). As we expected, pure tumor only mode was not able to discriminate a large portion of germline variants, resulting in very low precision. To better capture somatic variation in clinical tumor-only WES data, the inclusion of at least a small panel of normal samples should be mandatory.

### 3.3 External validation

The two main approaches that TOSCA uses to distinguish between somatic and germline mutations have been first validated by their authors in previous works. The ‘pure’ tumor-only mode leverages on extended rebuild of Sukhai *et al*. (2019) algorithm, which has been developed specifically for panel-based Next Generation Sequencing (NGS) testing. The authors proved that this algorithm is capable of defining somatic variants in a small-medium as well as large-sized targeted amplicon panel. On a medium panel of 48 gene, the proportion of true somatic variants correctly identified by the tumor-only filtration method out of all true somatic variants detected through matched blood samples (sensitivity) was about 100%, while the proportion of true germline variants correctly identified by the tumor-only filtration method out of all true germline variants detected through matched blood samples (specificity) was about 95.5%. With the use of a larger panel of 555 genes, the same algorithm obtained a sensitivity and specificity values that were only slightly reduced (99.6% and 86.9%, respectively). On the other side, when implemented in ‘hybrid’ mode with a pool of unmatched normal samples, TOSCA relies on tumor purity and ploidy estimation via R package PureCN, which has been shown to be a reliable method for somatic status predictions of variants also in clinical tumor-only WES data. Oh *et al*. (2020) used PureCN workflow with two different WES dataset obtaining sensitivity and specificity values of 96.1% and 88.1% in the first and 97.2% and 96.6% in the second dataset, respectively.

## 4 Conclusion

The TOSCA method leverages deep massively parallel sequencing to predict variant somatic and germline origin without a matched normal control. Since none algorithm can be considered 100% reliable, and also PureCN may return ambiguous values of purity and ploidy in a minority of cases of low purity or of high heterogeneity, we always recommended an additional step of manual correction when a classification of somatic variants is performed. While the definitive approach for discovery of novel somatic mutations includes sequencing data from a patient matched normal, TOSCA represents an open-source tool for functional ranking and interpretation of alterations discovered on routine testing performed with tumor alone and can enable assay development and clinical research. Furthermore, implementation of our workflow on Snakemake framework will facilitate users, even those with no coding experience, to process their own data easily.

## Funding

This work was supported by the Italian Association for Cancer Research (AIRC) [21198].

*Conflict of Interest*: none declared.

## Supplementary Material

vbac070_Supplementary_Data
