# Supplementary material for: TOSCA: an automated Tumor Only Somatic CAlling workflow for somatic mutation detection without matched normal samples
Source: Bioinform Adv. 2022 Sep 26;2(1):vbac070. doi: 10.1093/bioadv/vbac070 (PMC9710689; doi:10.1093/bioadv/vbac070)
Supplement: vbac070_Supplementary_Data [file vbac070_supplementary_data.zip › SupplementaryFile1.pdf]

Supplementary file 1a. Full description of different software implemented in TOSCA

- Reads first undergo quality control with FastQC and adapter trimming using Trimmomatic (Bolger M et al., 2014).
- BWA (Li H, 2013) is used to map reads to the human genome.
- TOSCA also includes additional quality control tools, namely Mosdepth (Pedersen S, 2018) Samtools stat (Danecek P et al., 2021) and BamStats04 (Lindenbau P, 2015) for genome-wide coverage detection and sequence data assessment.
- Duplicated reads are subsequently marked/removed with Picard MarkDuplicates (http://broadinstitute.github.io/picard/), while base quality scores are re-calibrated with GATK BaseRecalibrator (McKenna A et al., 2010).
- Genomic positions differing from reference sequence, i. e., SNPs and short Indels are then identified with Mutect2 tool (Benjamin D et al., 2019), which has been developed by the Broad Institute as part of GATK best practices. Besides being the only tool that works natively in a 'tumor-only' mode, several studies indicated Mutect2 as one of the most stable and accurate somatic caller (Cai L et al., 2016, Alioto, TS et al., 2015).
- GATK best practices performed by TOSCA include also further steps for artifacts and contamination detection (activated via *FilterMutectCalls* module of Mutect2) that might be introduced during sequencing and decrease confidence in variant calling.

Supplementary file 1b. The tumor-only filtration strategy inspired by a decision tree filtration algorithm developed by Sukhai *et al*. (2019)

- Phase Ia: Retained by criteria of quality pass. Read depth > cutoff* (250x suggested), VAF > cutoff* (5% suggested)
  Phase Ib: Retained by criteria of variant type (non-synonymous) and located in regions of interest (e.g. exons).
- Phase II. Variants retained after phase I were labeled as somatic if they were present in COSMIC database release version 92 (Forbes SA *et al*., 2015) or absent in the four germline population variant databases or present in no more than one at a minor allele frequency (MAF) of < 1%. If variants are present in more than two germline databases at MAF > 1e-6 they are marked as germline, even if they are also present in COSMIC database. The four germline database are the following:
  1. 1000 Genomes phase 3 (Siva N, 2008)
  2. Exome Sequencing Project (ESP; ESP6500SI-V2 data set of Exome Variant Server, National Heart, Lung, and Blood Institute Grand Opportunity Exome Sequencing Project, Seattle, WA; http://evs.gs.washington.edu/EVS)
  3. Exome Aggregation Consortium version r1 (ExAC) (Lek M *et al*., 2016)
  4. dbSNP build 154 (Sherry ST *et al*., 2001)
- Phase III. Variants retained after phase II were labeled as somatic if they were not present in ClinVar (National Center for Biotechnology Information ClinVar; 20210828; https://www.ncbi.nlm.nih.gov/clinvar) (Landrum MJ *et al*., 2016) databases with a benign or likely benign classification

  * The cutoffs can be set by the user in the configuration file

Default parameters implemented in TOSCA and values suggested by Sukhai et al. (2019)

- Minimum read depth to call a variant: 250X (100-250x suggested)
- Minimum variant allele frequency: 5% (1-5% suggested)

References

Alioto, T., Buchhalter, I., Derdak, S. et al. A comprehensive assessment of somatic mutation detection in cancer using whole-genome sequencing. Nat Commun 6, 10001 (2015). https://doi.org/10.1038/ncomms10001

Benjamin D, Sato T, Cibulskis K, et al. Calling Somatic SNVs and Indels with Mutect2. bioRxiv; 2019. DOI: 10.1101/861054.

Bolger AM, Lohse M, Usadel B. Trimmomatic: a flexible trimmer for Illumina sequence data, Bioinformatics, Volume 30, Issue 15, 1 August 2014, Pages 2114–2120, https://doi.org/10.1093/bioinformatics/btu170

Cai, L., Yuan, W., Zhang, Z. et al. In-depth comparison of somatic point mutation callers based on different tumor next-generation sequencing depth data. Sci Rep 6, 36540 (2016). https://doi.org/10.1038/srep36540

Danecek P, Bonfield JK, Liddle J, Marshall J, Ohan V, Pollard MO, Whitwham A, Keane T, McCarthy SA, Davies RM, Li H. Twelve years of SAMtools and BCFtools. Gigascience. 2021 Feb 16;10(2):giab008. doi: 10.1093/gigascience/giab008. PMID: 33590861; PMCID: PMC7931819.

Forbes SA, Beare D, Gunasekaran P, Leung K, Bindal N, Boutselakis H, Ding M, Bamford S, Cole C, Ward S, Kok CY, Jia M,De T, Teague JW, Stratton MR, McDermott U, Campbell PJ: COSMIC: exploring the world's knowledge of somatic mutations in human cancer. Nucleic Acids Res 2015, 43:D805eD811

Landrum MJ, Lee JM, Benson M, Brown G, Chao C, Chitipiralla S,Gu B, Hart J, Hoffman D, Hoover J, Jang W, Katz K, Ovetsky M,Riley G, Sethi A, Tully R, Villamarin-Salomon R, Rubinstein W, Maglott DR: ClinVar: public archive of interpretations of clinically relevant variants. Nucleic Acids Res 2016, 44:D862eD868

Lek M, Karczewski KJ, Minikel EV, Samocha KE, Banks E,Fennell T, et al: Analysis of protein-coding genetic variation in 60,706 humans. Nature 2016, 536:285e291

Li H. (2013) Aligning sequence reads, clone sequences and assembly contigs with BWA-MEM. arXiv:1303.3997v2

Lindenbaum, Pierre (2015): JVarkit: java-based utilities for Bioinformatics. Figshare. http://dx.doi.org/10.6084/m9.figshare.1425030

McKenna A, Hanna M, Banks E, Sivachenko A, Cibulskis K, Kernytsky A, Garimella K, Altshuler D, Gabriel S, Daly M, DePristo MA. The Genome Analysis Toolkit: a MapReduce framework for analyzing next-generation DNA sequencing data. Genome Res. 2010 Sep;20(9):1297-303. doi: 10.1101/gr.107524.110. Epub 2010 Jul 19. PMID: 20644199; PMCID: PMC2928508.

Pedersen BS, Quinlan AR. Mosdepth: quick coverage calculation for genomes and exomes. Bioinformatics. 2018 Mar 1;34(5):867-868. doi: 10.1093/bioinformatics/btx699. PMID: 29096012; PMCID: PMC6030888.

Sherry ST, Ward MH, Kholodov M, Baker J, Phan L, Smigielski EM, Sirotkin K: dbSNP: the NCBI database of genetic variation. Nucleic Acids Res 2001, 29:308e311

Siva N: 1000 Genomes project. Nat Biotechnol 2008, 26:256

Sukhai MA, Misyura M, Thomas M, Garg S, Zhang T, Stickle N, Virtanen C, Bedard PL, Siu LL, Smets T, Thijs G, Van Vooren S, Kamel-Reid S, Stockley TL. Somatic Tumor Variant Filtration Strategies to Optimize Tumor-Only Molecular Profiling Using Targeted Next-Generation Sequencing Panels. J Mol Diagn. 2019 Mar;21(2):261-273. doi: 10.1016/j.jmoldx.2018.09.008. Epub 2018 Dec 19. PMID: 30576869.
